# Characterization of novel pneumatic mixing for single-use bioreactor application

**DOI:** 10.1186/1753-6561-5-S8-O12

**Published:** 2011-11-22

**Authors:** Brian Lee, David Fang, Matthew Croughan, Manuel Carrondo, Sang-Hoon Paik

**Affiliations:** 1 PBS Biotech Inc., Camarillo, California, 93012, USA; 2 Systems QbD, Newbury Park, California, 91320, USA; 3 Keck Graduate Institute (KGI), Claremont, California, 91711, USA; 4 Instituto de Biologia Experimental e Tecnologica (IBET), Lisbon, 2780-157, Portugal; 5 Green Cross Corp., Yongin-Si, KyungGi-Do, 449-070, South Korea

## Background

The novel bioreactor system from PBS Biotech^®^ is the first with a pneumatic mixing device powered solely by gas buoyancy, eliminating the need for an external mechanical agitator. The patented design of the Air-Wheel mixing device promotes not only high tangential fluid flow around the wheel but also efficient radial and axial flows. The leverage effect of the Air-Wheel mixing device also allows for lower gassing requirement (v/v) with increasing working volume, making the power utilization from the gas buoyancy more efficient with scale.

A series of physical tests were performed to demonstrate that PBS systems can promote uniform, homogenous mixing over a wide range of working volumes and can offer a low-shear environment for cell culture. A series of biological tests were then performed at various evaluation sites to confirm the physical test results.

## Results

Mixing time was calculated in PBS systems ranging from 2L to 5,000L working volume by measuring the change in conductivity readings from bolus additions of concentrated salt solution. 95% mixing times were found to be between 20 sec and 62 sec over this range of working volumes in the PBS systems (Table 1), significantly shorter than reported values in conventional stirred tank systems.

**Table 1:** 95% mixing time from 2L to 5,000L working volumes in PBS prototype units

	2L	10L	50L	250L	1,000L	5,000L
Gas Flow Rate (VVM)	0.07	0.06	0.05	0.04	0.03	0.02
Mixing Time (sec)	20	25	30	38	53	62

Shear stress and turbulent kinetic energy dissipation rate (ε, in m^2^/s^3^) were calculated from computational fluid dynamics (CFD) modeling on Star CCM+ software. Lower average shear stress (τ_avg,_ in Pa) on the impeller and lower average energy dissipation rate (ε, in m^2^/s^3^) in the impeller region were found in the PBS system compared to stirred-tank bioreactors at typical impeller speeds at the respective working volumes. Comparably low level of shear stress (<0.5 Pa) was found in the PBS system ranging from 3L to 2,500L working volume, and the uniformity of mixing in the PBS system was confirmed with the consistency of ε (<0.01 m^2^/s^3^) over the same broad range of working volumes.

At Keck Graduate Institute, lactate-adapted CHO DG44 cells were cultured in fed-batch mode in 3L and 15L PBS systems using CD OptiCHO^TM^ medium from Invitrogen^TM^. Comparable peak viable cell density of 25x10^6^ cell/mL was attained in both systems, and >90% viability was maintained through 12 days of culture.

At IBET, Sf9 insect cells were infected with baculovirus expressing enhanced green fluorescent protein (eGFP) in a 3L PBS system. Infection with the baculovirus was performed at MOI of 0.8 on day 1 of the run. Successful expansion of baculovirus in Sf9 cells was demonstrated by high level of eGFP expression and infected particles detected after day 1.

**Figure 1:**
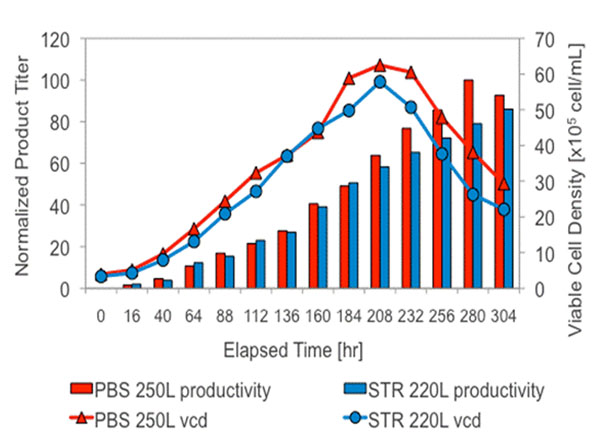
Viable cell density and product titer comparison between 250L PBS bioreactor and 220L stainless steel bioreactor for an Fc-fusion protein

At Green Cross Corporation, a process for producing Fc-fusion protein was run in the 250L PBS system in parallel with the 300L (220L w.v.) stirred-tank bioreactor in fed-batch mode over a span of 19 days. Superior peak viable cell density and product titer were achieved in the PBS system compared to the stirred-tank system (Figure 1). Protein physical methods (reduced PAGE and IEF), ELISA, and in-vitro bioactivity assay (MTS cell inhibition assay) revealed equivalence of products made in PBS and stirred-tank systems.

## Conclusions

CFD modeling results revealed uniform, homogenous mixing pattern with low-shear stress in PBS systems over a wide range of working volumes. Short mixing times from conductivity-based mixing tests confirmed the efficiency of mixing in PBS systems.

Recent biological studies performed at beta test sites further demonstrated successful performance in three key tests:

1) Scalable growth for a high cell density process in 3L and 15L PBS systems,

2) Successful baculovirus expansion in insect cells in the 3L PBS system, and

3) Comparable growth and product performance in the 250L PBS system against a 220L stirred-tank bioreactor using a Fc-fusion protein-producing process.

